# The pH-Responsive Transcription Factor PacC Governs Pathogenicity and Ochratoxin A Biosynthesis in *Aspergillus carbonarius*

**DOI:** 10.3389/fmicb.2020.00210

**Published:** 2020-02-13

**Authors:** Omer Barda, Uriel Maor, Sudharsan Sadhasivam, Yang Bi, Varda Zakin, Dov Prusky, Edward Sionov

**Affiliations:** ^1^Institute of Postharvest and Food Sciences, Agricultural Research Organization, Volcani Center, Rishon LeZion, Israel; ^2^Institute of Biochemistry, Food Science and Nutrition, The Robert H. Smith Faculty of Agriculture, Food and Environment, The Hebrew University of Jerusalem, Rehovot, Israel; ^3^College of Food Science and Engineering, Gansu Agricultural University, Lanzhou, China

**Keywords:** *Aspergillus carbonarius*, PacC, pH regulation, OTA biosynthesis, post-harvest disease

## Abstract

Pathogenic fungi must respond effectively to changes in environmental pH for successful host colonization, virulence and toxin production. *Aspergillus carbonarius* is a mycotoxigenic pathogen with the ability to colonize many plant hosts and secrete ochratoxin A (OTA). In this study, we characterized the functions and addressed the role of PacC-mediated pH signaling in *A. carbonarius* pathogenicity using designed *pacC* gene knockout mutant. Δ*AcpacC* mutant displayed an acidity-mimicking phenotype, which resulted in impaired fungal growth at neutral/alkaline pH, accompanied by reduced sporulation and conidial germination compared to the wild type (WT) strain. The Δ*AcpacC* mutant was unable to efficiently acidify the growth media as a direct result of diminished gluconic and citric acid production. Furthermore, loss of *AcpacC* resulted in a complete inhibition of OTA production at pH 7.0. Additionally, Δ*AcpacC* mutant exhibited attenuated virulence compared to the WT toward grapes and nectarine fruits. Reintroduction of *pacC* gene into Δ*AcpacC* mutant restored the WT phenotype. Our results demonstrate important roles of PacC of *A. carbonarius* in OTA biosynthesis and in pathogenicity by controlling transcription of genes important for fungal secondary metabolism and infection.

## Introduction

In order to survive and proliferate under diverse conditions, fungal microorganisms must be able to sense and respond to rapidly changing environmental stresses (Prusky et al., 2013). Fungal pathogens are not only able to survive in a diverse range of environmental conditions, but also have evolved abilities to recognize, penetrate and attack their hosts, while responding to chemical and physical signals from the host. Ambient pH is one of these environmental conditions and acts as an important signal for fungal growth, development, secondary metabolism and host infection (Drori et al., 2003; Li et al., 2010; Vylkova et al., 2011; Barad et al., 2014, 2016). *Aspergillus carbonarius* is frequently responsible for post-harvest decay of various fresh fruits, including grapes, peaches, pears, citrus and nectarines (JECFA, 2001). In addition to its pathogenicity, *A. carbonarius* is also considered as the main producer of ochratoxin A (OTA) – a potent nephrotoxin which may exhibit carcinogenic, teratogenic and immunotoxic properties in animals and possibly in humans (IARC, 1993; Pfohl-Leszkowicz and Manderville, 2007). Our recent study demonstrated that ambient pH plays an important role in *A. carbonarius* pathogenicity and OTA biosynthesis (Maor et al., 2017). Secretion of gluconic acid (GLA) by *A. carbonarius* caused direct fruit tissue acidification and induced accumulation of OTA in colonized grapes. Previous findings indicated that acidification of the apple fruit host environment through secretion of organic acids enhanced maceration and colonization of the fruit by *Penicillium expansum* (Prusky et al., 2004; Barad et al., 2012). Barad et al. (2014) pointed out the importance of the acidification process driven by GLA production in the activation of patulin biosynthesis and its contribution to the enhanced pathogenicity of *P. expansum* in apples.

Ambient pH signaling in filamentous fungi was first discovered in *Aspergillus nidulans*; it’s mediated by transcription factor PacC and six Pal proteins (PalA, PalB, PalC, PalF, PalH, and PalI), which may regulate both acid- and alkaline-expressed genes in several fungal species (Caddick et al., 1986; Tilburn et al., 1995; Penalva et al., 2008; Ment et al., 2015). The external pH signal is transmitted by fungal signaling pathway from the extracellular environment to the nucleus, where it regulates the expressions of PacC dependent genes, which are involved in secondary metabolism, cell wall biosynthesis and fungal pathogenesis (Penalva and Arst, 2002; You et al., 2007; Luo et al., 2017). PacC has been previously shown to differentially regulate virulence as well as biosynthesis and secretion of secondary metabolites in several fungal species. Decreased fungal virulence as well as reduction in biosynthesis of secondary metabolites, as a result of *pacC* knockout, have been observed in several *Aspergillus* and *Penicillium* species (Suarez and Penalva, 1996; Keller et al., 1997; Bergh and Brakhage, 1998; Zhang et al., 2013; Chen et al., 2018; Wang et al., 2018). In contrast, disruption of *pacC* resulted in increased pathogenicity of *Fusarium oxysporum* (Caracuel et al., 2003) and higher trichothecene and fumonisin production in *F. graminearum* and *Fusarium verticillioides*, respectively, compared to the wild type strains (Flaherty et al., 2003; Merhej et al., 2011), suggesting that this transcription factor acts as a negative regulator of virulence and secondary metabolism in *Fusarium* spp.

It has been already reported that OTA biosynthesis is regulated by PacC in *Aspergillus ochraceus* (Wang et al., 2018). Although the growth of the Δ*AopacC* mutant and its ability to produce OTA were compromised to some degree, an increase in conidia formation has been observed compared to that of the wild type strain, suggesting that PacC in *A. ochraceus* positively regulates growth and OTA biosynthesis, but has a negative regulatory role in sporulation. In the present study we investigated the role of the pH regulatory factor PacC in virulence and OTA production in *A. carbonarius*, which is one of the most important mycotoxigenic pathogens. *AcpacC* gene deletion showed the significance of this transcription factor in germination, sporulation, mycelial growth, OTA biosynthesis and virulence in fruits. Our results suggest that AcPacC is a positive regulator of virulence in *A. carbonarius* by mediating expression of the glucose oxidase encoding gene (*gox*) and the genes encoding for cell wall degrading enzymes.

## Materials and Methods

### Fungal Strain and Growth Conditions

The wild type (WT) strain *A. carbonarius* NRRL 368 was obtained from USDA Agricultural Research Service Culture Collection (Northern Regional Research Laboratory, Peoria, IL, United States). The WT strain and mutants generated in this study were grown at 28°C and maintained on Potato Dextrose Agar (PDA) plates (BD, Franklin Lakes, NJ, United States). Conidia were harvested and adjusted using a haemocytometer to the indicated concentrations.

### Gene Knockout and Complementation

Construction of the *AcpacC* gene replacement plasmid was achieved by PCR-amplifying genomic flanking regions using specific primer pairs that incorporated a single 2-deoxyuridine nucleoside near the 5′ ends (primers U-f1 × U-r1 for the promoter region and primers D-f1 × D-r1 for the terminator region). Both DNA fragments and the pre-digested pRFHU2 binary vector (Frandsen et al., 2008) were mixed together and were treated with the USER enzyme (New England Biolabs, Ipswich, MA, United States) to obtain the plasmid pRFHU2-AcpacC. An aliquot of the mixture was used directly in chemical transformation of high-efficiency *Escherichia coli* DH5α cells (New England Biolabs, Ipswich, MA, United States) without prior ligation. Kanamycin resistant transformants were screened by PCR for validation of proper fusion events. Then, the plasmid was introduced into electro-competent *Agrobacterium tumefaciens* AGL-1 cells.

A single colony of *A. tumefaciens* AGL-1 carrying plasmid pRFHU2-AcpacC was used to inoculate a starter culture and incubated for 24 h. Bacterial cells were centrifuged, washed with induction medium (10 mM K_2_HPO_4_, 10 mM KH_2_PO_4_, 2.5 mM NaCl, 2 mM MgSO_4_, 0.6 mM CaCl_2_, 9 μM FeSO_4_, 4 mM (NH_4_)_2_SO_4_, 10 mM glucose, 40 mM MES pH 5.3, 0.5% glycerol) and diluted in the same medium amended with 200 μM acetosyringone (IMAS) to an OD600 = 0.15. Cells were grown at 28°C and 200 rpm until they reached an OD600 = 0.75. Equal volumes of IMAS induced bacterial culture and conidial suspension of *A. carbonarius* (10^6^ conidia/ml) were mixed and spread onto Whatman filter papers, which were placed on agar plates containing the co-cultivation medium (same as IMAS, but containing 5 mM instead of 10 mM of glucose). After co-cultivation at 28°C for 48 h, the membranes were transferred to PDA plates containing hygromycin B (100 μg/mL) as the selection agent for fungal transformants, and cefotaxime (200 μg/mL) to inhibit growth of *A. tumefaciens* cells. Hygromycin resistant colonies appeared after 3–4 days of incubation at 28°C. Disruption of *AcpacC* was confirmed by PCR analyses of the transformants.

A restriction free cloning method (van den Ent and Lowe, 2006) was employed in order to replace the hygromycin resistance gene *hph* of the pRFHU2 vector with the phleomycin resistance gene *ble* amplified from the pBC-Phleo plasmid (Silar, 1995) using primer pair RFC-f1 x RFC-r1. Proper substitution of the resistance genes was confirmed by DNA sequencing and the new plasmid was termed pRFPU2. For the construction of the complementation vector, two genomic fragments consisting of the entire *AcpacC* cassette (primer pair U-f1 × U-r2) and the gene’s terminator region (primer pair D-f1 × D-r1), were USER cloned to the pre-digested pRFPU2 vector to generate pRFPU2-AcpacC-c as described before. Conidia of the Δ*AcpacC* knockout strain were transformed using *A. tumefaciens* AGL-1 cells carrying the plasmid pRFPU2-AcpacC-c as described before. For selecting phleomycin resistant complementation transformants, phleomycin (50 μg/mL) was enough to prevent growth of untransformed conidia. Analysis of transformants for reintroduction of the endogenous *AcpacC* cassette was done by PCR. All primers used to create and confirm the mutant and complement strains are listed in Supplementary Table S1.

### Physiological Analysis

Radial growth and sporulation (conidial production) were assessed on solid YES media (20 g bacto yeast extract, 150 g sucrose and 15 g bacto agar per liter) adjusted with 10 M HCl or 10 M NaOH to pH 4.0, 7.0 and 8.0, while conidial germination was evaluated in YES broth media (20 g bacto yeast extract and 150 g sucrose per liter) adjusted to pH 4.0 and pH 7.0.

For radial growth assessment, 90 mm agar plates were point inoculated with 10^2^ conidia of either the WT, knockout or complement strain and incubated at 28°C. Growth was monitored by diameter measurements on a daily basis for 10 days using three replicate plates per strain.

For conidial production quantification, 55 mm agar plates containing 10^5^ conidia of each strain were incubated at 28°C for 7 days. To accurately count conidia, two 1 cm plugs from each plate were homogenized in 3 ml water containing 0.01% Tween 20, diluted and counted with a haemocytometer. Conidial production was quantified starting by the 3rd day post-inoculation using three replicate plates per strain.

For germination evaluation, the conidia concentration of all strains was adjusted to 10^4^ conidia/ml in the medium. 0.5 ml of each conidial suspension was distributed into three replicate wells of a 24-well sterile culture plate (SPL Life Sciences, Pocheon, South Korea). Time-course microscopy was carried out over 24 h at 28°C using a Nikon Eclipse T*i* inverted microscope (Nikon, Tokyo, Japan) equipped with a ProScan motorized XY stage (Prior Scientific, Cambridge, United Kingdom) with a LAUDA ECO RE 415 temperature-controlled incubator (LAUDA-Brinkmann, Delran, NJ, United States). Images were captured at 1-h intervals, beginning 2 h post-incubation using an ANDOR zyla 5.5 MP sCMOS camera (Andor Technology, Belfast, Northern Ireland) and processed using the NIS elements AR 4.6 (64 bit) software package. Conidia were considered to have germinated when germ tubes arose from the swollen conidial base. Number of conidia germlings were counted for each strain and the percent of germinated conidia was plotted against time.

### pH Measurements, Organic Acids and OTA Analysis

A 10^6^ fungal conidia/ml solution (100 μl) was inoculated onto 55 mm petri dishes containing 10 ml of solid YES media adjusted to pH 4.0 or pH 7.0. The plates were incubated at 28°C in the dark for 2–13 days as needed for sample collection.

pH was measured directly in the agar cultures with a double pore slim electrode connected to a Sartorius PB-11 Basic Meter (Sartorius, Göttingen, Germany).

For assessment of organic acids production, five 1-cm diameter discs of agar were placed in 5 ml of sterilized water and crushed to homogeneity. A 1 ml aliquot of the solution was sampled in a 1.5 ml microcentrifuge tube and centrifuged for 10 min at 20,800 *g*. The supernatant was taken for GLA and citric acid analysis using test kits applying enzymatic methods for the specific measurement of total D-Gluconic acid and citric acid contents (Megazyme, Wicklow, Ireland) according to the manufacturer’s instructions.

To evaluate OTA levels, five 1-cm diameter discs of agar were added to 1.7 ml of HPLC grade methanol (Bio-Lab, Jerusalem, Israel) and crushed to homogeneity. OTA was extracted by shaking for 30 min at 150 RPM on an orbital shaking platform and centrifuged for 10 min at 20,800 *g*. The supernatant was filtered through a 0.22 μm PTFE syringe filter (Agela Technologies, Tianjin, China) and kept at −20°C prior to HPLC analysis. OTA was quantitatively analyzed by injection of 20 μl into a reverse phase UHPLC system (Waters ACQUITY Arc, FTN-R, Milford, MA, United States). The mobile phase consisted of acetonitrile:water:acetic acid (99:99:2, v/v/v) at 0.5 ml/min through a Kinetex 2.6 μm XB-C18 (100 × 2.1 mm) with a security guard column C18 (4 × 2 mm) (Phenomenex, Torrance, CA, United States). The column temperature was maintained at 30°C. The OTA peak was detected with a fluorescence detector (excitation at 330 nm and emission at 450 nm) and quantified by comparing with a calibration curve of the standard mycotoxin (Fermentek, Jerusalem, Israel).

### Colonization and Pathogenicity Experiments

“Zani” seedless grapes and “Sun Snow” nectarines were obtained from a local supermarket. Fruits were subjected to surface sterilization using 1% sodium hypochlorite solution for 1 min, and immediately rinsed in sterile distilled water. A 10 μl conidial suspension containing 10^6^ conidia/ml of either the WT, the Δ*AcpacC* mutant strain or the *AcpacC*-c complement strain was injected directly into the sterilized fruits at 2 mm depth. Following inoculation, the fruits were incubated in covered plastic containers at 28°C for 2–9 days as needed for symptom monitoring and sample collection, and the diameters of the rotten spots were recorded daily.

The pH of nectarine tissues was measured by inserting a double pore slim electrode directly into the tested area. To analyze GLA content in inoculated nectarine fruits, 1.7 gr of the macerated necrotic area were taken, 5 ml of sterilized water were added and the tissues were homogenized. A 1 ml aliquot of the solution was sampled in a 1.5 ml microcentrifuge tube, centrifuged for 10 min at 20,800 *g*, and the amounts of GLA produced were measured as described above.

For OTA analysis in colonized grapes and nectarines, 1.7 gr of the macerated necrotic area were taken, 1.7 ml of HPLC grade methanol (Bio-Lab, Jerusalem, Israel) were added and the tissues were homogenized. Then, OTA was quantitatively analyzed as described above.

### RNA Extraction and qRT-PCR Analysis of Gene Transcription Profile

Mycelia from the *in vitro* experiments were harvested at the appropriate time, weighed, frozen in liquid nitrogen, lyophilized for 24 h and kept at −80°C until use. In colonized nectarines, mycelia containing exocarp (peel) was removed, frozen in liquid nitrogen and lyophilized prior to RNA extraction. Total RNA was extracted from 100 mg of lyophilized tissue of the selected samples using the Hybrid-R RNA isolation kit (GeneAll, Seoul, South Korea) according to the manufacturer’s protocol. The DNase and reverse-transcription reactions were performed on 1 μg of total RNA with the Maxima First-Strand cDNA Synthesis Kit (Thermo Scientific, Waltham, MA, United States) according to the manufacturer’s instructions. The cDNA samples were diluted 1:10 (v/v) with ultrapure water. The quantitative real-time PCR was performed using Fast SYBR green Master Mix (Applied Biosystems, Waltham, MA, United States) in a StepOnePlus Real-Time PCR System (Applied Biosystems, Waltham, MA, United States). The PCR conditions were as follows: 95°C for 20 s, followed by 40 cycles of 95°C for 3 s and 60°C for 20 s. The samples were normalized using β*-tubulin* as endogenous control and the relative expression levels were measured using the 2^(–ΔΔCt)^ analysis method. Results were analyzed with StepOne software v2.3. Primer sequences used for qRT-PCR analysis are listed in Supplementary Table S2.

### Statistical Analysis

Student’s *t*-test was performed when data was normally distributed and the sample variances were equal. For multiple comparisons, one-way ANOVA was performed when the equal variance test was passed. Significance was accepted at *p* < 0.05. All experiments described here are representative of at least three independent experiments with the same pattern of results.

## Results and Discussion

### Creation and Validation of *pacC* Deletion and Complementation Strains of *A. carbonarius*

In order to explore the functional roles of PacC in the physiology and pathogenicity of *A. carbonarius*, *AcpacC* deletion and complementation strains were generated. For *AcpacC* deletion, a targeted gene deletion strategy was employed using *A. tumefaciens* mediated transformation of *A. carbonarius* NRRL 368 WT strain (Supplementary Figure S1). Gene replacement plasmid, pRFHU2-AcpacC, was obtained by a USER Friendly cloning system (Frandsen et al., 2008). Co-cultivation of *A. tumefaciens* cells carrying pRFHU2-AcpacC with the conidia of *A. carbonarius* led to the appearance of hygromycin B-resistant colonies approximately 4 days after transfer to the selective PDA plates. Disruption of *AcpacC* was confirmed by several PCR analyses for the introduction of the hygromycin resistance gene coding sequence, correct genomic placement of the 5′ and 3′ flanking sequences and the absence of the *AcpacC* sequence. With respect to the *AcpacC* complement strain, twelve single transformants were initially selected on PDA medium supplemented with phleomycin (50 μg/ml). The phleomycin resistant strains were diagnosed by PCR to confirm the integration of the WT allele in the Δ*AcpacC* strain using the same set of primers that amplify the *AcpacC* ORF. As expected, the *AcpacC-c* strain revealed the expected band of 472 bp (Supplementary Figure S2). One of the correct *AcpacC-c* strains was used for the following experiments.

### AcPacC Is Required for Fungal Growth, Conidial Formation and Germination

**FIGURE 1:**
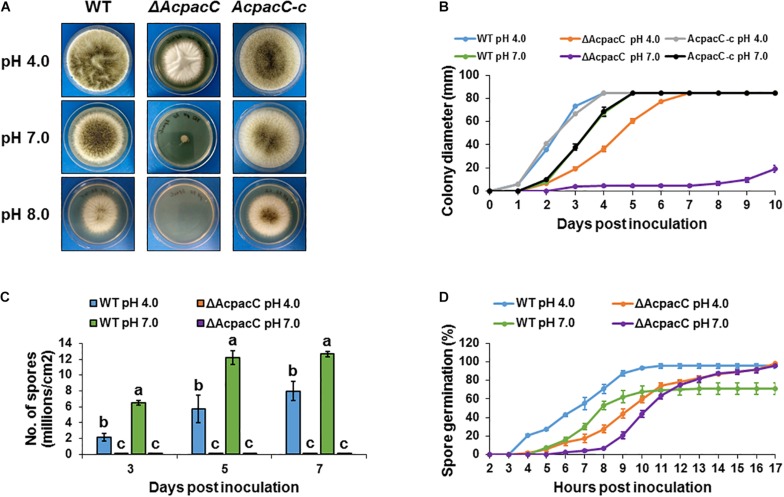
Physiological analyses of the WT and mutant strains of *A. carbonarius* at different pH conditions. **(A)** Growth phenotype and **(B)** radial growth of the WT, Δ*AcpacC* and *AcpacC-c* strains on solid YES media at 28°C under pH 4.0, 7.0, and 8.0. **(C)** Conidiation of the WT and Δ*AcpacC* strains on solid YES media at pH 4.0 and 7.0. **(D)** Germination rates in the WT and Δ*AcpacC* strains were assessed in static YES broth media at 28°C under pH 4.0 and 7.0. Error bars represent the standard error of the mean (SEM) across three independent replicates. Different letters above the columns indicate statistically significant differences at *p* < 0.05, as determined using the Tukey’s honest significant difference test.

**FIGURE 2:**
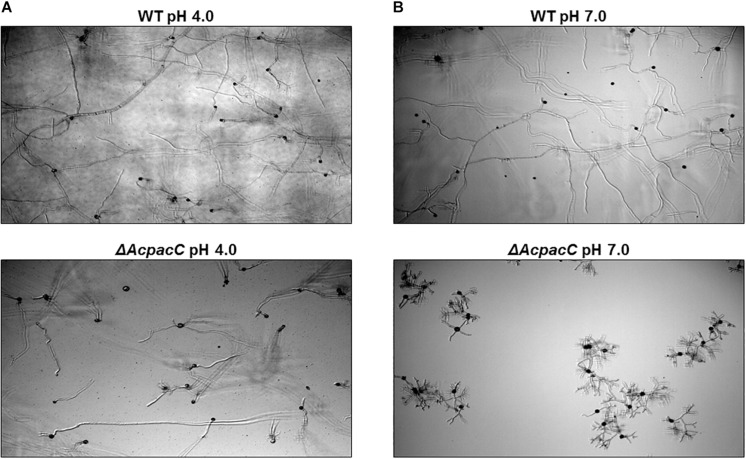
Microscopic observation of hyphal morphology of WT and Δ*AcpacC* strains. Images of time-course microscopy were captured 17 h following incubation of WT and Δ*AcpacC* conidia suspensions in YES broth media adjusted to pH 4.0 **(A)** and pH 7.0 **(B)**.

Physiological analysis revealed that compared to the WT strain the growth of the Δ*AcpacC* mutant was reduced on YES synthetic media under acidic condition at pH 4.0, significantly impaired at pH 7.0 and completely inhibited under alkaline condition at pH 8.0 (Figures 1A,B). Due to the inability of the mutant strain to grow in alkaline conditions, the following experiments were performed under acidic and neutral pH conditions. Conidia production in Δ*AcpacC* mutant strain was severely inhibited at all examined time points under both acidic and neutral pH conditions (Figure 1C). Our study demonstrated that pH, as an environmental factor, may affect conidial germination of *A. carbonarius*. Germination rate of *A. carbonarius* was inhibited by 29% under ambient pH 7.0 (Figure 1D). It has been previously reported that germination of *P. expansum* conidia was inhibited significantly under ambient pH 2.0 and 8.0, probably through impairing protein synthesis and folding (Li et al., 2010). Conidial germination in the Δ*AcpacC* mutant strain was delayed compared to the WT at both pH conditions, although 100% germination of these conidia has been observed following 17 h of incubation (Figure 1D). By this time point, the ability of the mutant strain to develop hyphae in liquid culture at acidic pH was normal as was the ability to maintain hyphal growth (Figure 2A). In contrast, at pH 7.0 hyphal formation and growth of Δ*AcpacC* strain was significantly stunted and characterized by severe hyper-branching compared to the WT (Figure 2B). Normal growth, sporulation, conidial germination and hyphal formation were recovered in the *AcpacC-c* strain, indicating that AcPacC is required for morphogenetic development of *A. carbonarius*, especially in alkaline conditions.

Similarly to Δ*AcpacC*, growth of the *pacC* disrupted mutants in *Sclerotinia sclerotiorum*, *F. oxysporum*, *Colletotrichum acutatum*, *F. graminearum* and *Penicillium digitatum* was slightly reduced under acidic pH but severely impaired under alkaline conditions (Caracuel et al., 2003; Rollins, 2003; You et al., 2007; Merhej et al., 2011; Zhang et al., 2013). However, deletion of *pacC* showed poor growth phenotype under both acidic and alkaline pH conditions in *P. expansum*, *Metarhizium robertsii* and *Ganoderma lucidum* (Huang et al., 2015; Wu et al., 2016; Chen et al., 2018). Interestingly, *A. ochraceus* Δ*pacC* mutant strain had slightly impaired growth under alkaline conditions, but similar growth rate to the WT in acidic pH. On the contrary, unlike Δ*AcpacC* strain, an increase in conidia formation was observed in *A. ochraceus* Δ*pacC* mutant compared to that of the WT strain under all the pH conditions (Wang et al., 2018). Therefore, it is likely that PacC plays different roles in mycelial growth and sporulation of different fungal pathogens.

### AcPacC Regulates Production of Organic Acids in *A. carbonarius*

**FIGURE 3:**
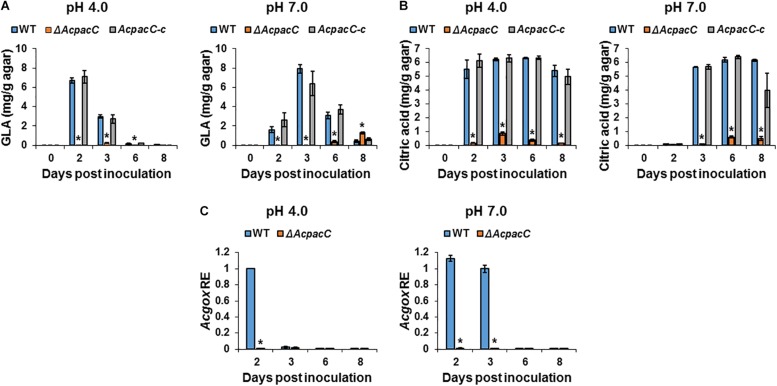
Effects of AcPacC on organic acids production in *A. carbonarius*. Gluconic acid (GLA) **(A)** and citric acid **(B)** accumulation by the WT, Δ*AcpacC* and *AcpacC-c* strains under different pH conditions. **(C)** Differential expression of the *Acgox* gene between WT and Δ*AcpacC* at pH 4.0 and 7.0. Average values of three replicates (± standard error) are reported. Experiments were repeated three times and results of a single representative experiment are shown. Asterisks denote significant differences between strains at *p* < 0.05.

Post-harvest fungal pathogens were reported to enhance their virulence by locally modulating the host’s ambient pH (Prusky et al., 2014). Previous studies have shown that *Penicillium* spp. (*P. expansum*, *P. digitatum*) and *A. carbonarius* acidify the ambient environments of deciduous fruits during decay development by secretion of significant amounts of organic acids, mainly citric and gluconic acids (Prusky et al., 2004; Barad et al., 2012, 2014; Maor et al., 2017). Gluconic acid accumulation by *P. expansum* and *A. carbonarius* is pH-dependent and is mainly regulated by glucose oxidase (GOX) that catalyzes the oxidation of glucose to gluconic acid. In the current study, a significant decrease in the formation of both citric and gluconic acids was observed in Δ*AcpacC* knockout mutant compared with the WT under acidic and neutral pH conditions at all tested time points (Figures 3A,B). The kinetics of the gene transcript level shows that *Acgox* gene expression was markedly down-regulated in Δ*AcpacC* in both acidic and neutral pH conditions, compared to the WT strain (Figure 3C), indicating that AcPacC directly regulates gluconic acid production by positive modulation of *A. carbonarius* glucose oxidase-encoding gene. In *P. expansum*, two *pacC*-RNAi mutants with downregulation of PacC (silenced by RNAi technology) resulted in a 63 and 27% reduction in gluconic acid production, respectively (Barad et al., 2014). This relatively moderate reduction could be attributed to residual PacC expressions in *pacC*-RNAi mutants. A recent study reported that GOX, which was identified by proteome analysis as an alkaline-expressed protein, is directly regulated by *P. expansum* transcription factor PacC (Chen et al., 2018). At acidic pH, the PacC protein is inactive and therefore unable to bind to the promoter sites of the target genes, however, under alkaline conditions PacC acts as an activator of alkaline-expressed genes and as a repressor of acid-expressed genes (Penalva et al., 2008).

### OTA Biosynthesis in *A. carbonarius* Is Regulated by AcPacC

**FIGURE 4:**
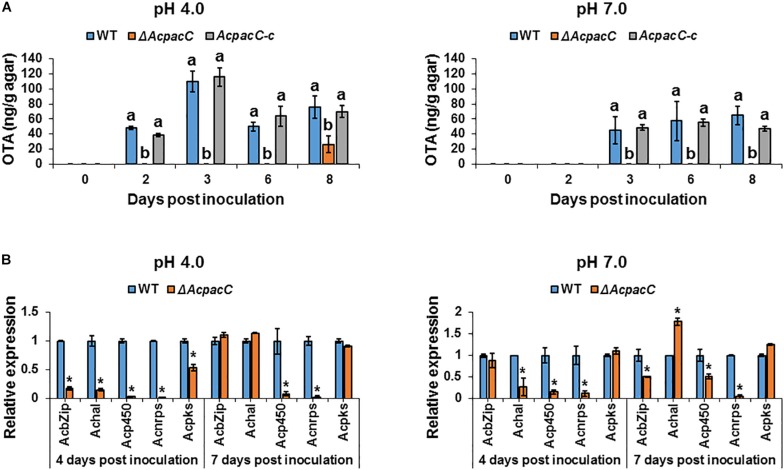
Effects of AcPacC on OTA biosynthesis in *A. carbonarius* at different pH conditions. **(A)** OTA production by the WT, Δ*AcpacC* and *AcpacC-c* strains at pH 4.0 and pH 7.0. **(B)** Relative expression of OTA cluster genes in WT and Δ*AcpacC* at days 4 and 7 post-inoculation. Relative expression was normalized using β*-tubulin* as an internal control. Average values of three replicates (± standard error) are presented. Experiments were repeated three times and results of a single representative experiment are shown. Different letters above the columns indicate statistically significant differences at *p* < 0.05, as determined using the Tukey’s honest significant difference test. Asterisks denote significant differences between strains at *p* < 0.05.

Among black aspergilli, *A. carbonarius* has shown a consistent ability to produce OTA and is the most probable source of this mycotoxin in a wide range of foods. Our previous study showed a clear pattern of pH modulation through secretion of organic acids by *A. carbonarius*, which acidify the ambient environment and induce OTA production in culture (Maor et al., 2017). In addition, Barad et al. (2014) demonstrated that down-regulation of *gox* gene in *P. expansum* (using RNAi technology) resulted in impaired ability to produce gluconic acid, which was accompanied by down-regulation in the relative expression of *idh* gene (encodes the isoepoxydon dehydrogenase enzyme, a key enzyme in the patulin biosynthesis pathway) and reduction in patulin accumulation. As shown in Figure 4A, OTA production by WT strain was significantly higher throughout the experiment under acidic condition, at pH 4.0, compared to the accumulation at pH 7.0. Deletion of *AcpacC* resulted in complete inhibition of OTA production at both pH 4.0 and 7.0 during the first 6 days after inoculation (Figure 4A). Nevertheless, under acidic condition a small amount of OTA was secreted by Δ*AcpacC* at day 8 of the experiment (Figure 4A). These results indicated that AcPacC is an important regulator in OTA biosynthesis in *A. carbonarius* under different pH conditions. Our findings suggest that not only the organic acids production could influence the accumulation of OTA, but also low pH itself might stimulate the mycotoxin biosynthesis. Moreover, we investigated the differential expression of all the five OTA biosynthetic cluster genes in Δ*AcpacC* and WT strains at ambient pH 4.0 and 7.0. As shown in Figure 4B, expressions of all five genes at day 4 of the experiment were down-regulated in Δ*AcpacC* at acidic pH. Under this condition, the relative expression of bZIP transcription factor, halogenase (HAL) and polyketide synthase (PKS) encoding genes in Δ*AcpacC* was similar to that of the WT strain at day 7 post-inoculation (Figure 4B); apparently, this is reflected in OTA production by the mutant at day 8 of the experiment. Although Δ*AcpacC* lost the ability of OTA production under neutral condition, the expression levels of several genes in the mutant strain, encoding bZIP transcription factor, PKS or HAL, were either unaffected or upregulated at pH 7.0 compared to the WT strain (Figure 4B), suggesting that these genes might be regulated by other transcription factors. PacC may act either as a positive or negative regulator of secondary metabolites biosynthesis (Brakhage, 2013). Similar to our findings, PacC was found to serve as a positive regulator of penicillin synthesis in *A. nidulans* and patulin biosynthesis in *P. expansum* (Bergh and Brakhage, 1998; Barad et al., 2014; Chen et al., 2018). On the contrary, PacC negatively regulates fumonisin biosynthesis in *F. verticillioides* and trichothecene biosynthesis in *F. graminearum* (Flaherty et al., 2003; Merhej et al., 2011). In *A. ochraceus*, another OTA producing pathogen, PacC played a positive role in regulating OTA biosynthesis, which was slightly impaired in *AopacC* loss-of-function mutant (Wang et al., 2018). Thus, PacC appears to function differently in regulating secondary metabolites in different fungal pathogens; yet, many unanswered questions remain on the mechanism of this regulation.

### PacC Is Required for Pathogenicity in *A. carbonarius* and OTA Contamination of Deciduous Fruits

**FIGURE 5:**
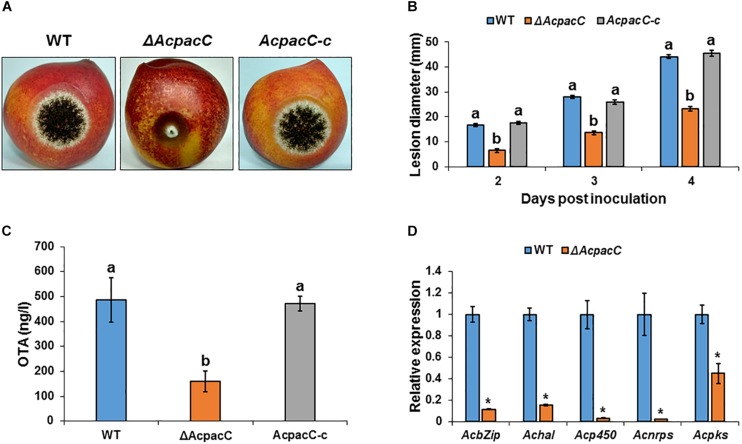
Effects of AcPacC on pathogenicity of *A. carbonarius* and OTA production in nectarines. **(A)** Disease symptoms on nectarine fruits inoculated with conidia of WT, Δ*AcpacC* and *AcpacC-c* strains at 3 days after inoculation. **(B)** Histogram showing the diameters of the rotten spots on infected nectarines. **(C)** OTA accumulation in infected nectarines, and **(D)** relative expression of OTA cluster genes in WT and Δ*AcpacC* strains. RNA was extracted from infected nectarines at day 4 post-inoculation. Relative expression was normalized using β*-tubulin* as an internal control. Error bars represent standard error of three independent biological replicates. Different letters above the columns indicate statistically significant differences at *p* < 0.05, as determined using the Tukey’s honest significant difference test. Asterisks denote significant differences between strains at *p* < 0.05.

Colonization of “Sun Snow” nectarines and white “Zani” grape berries by Δ*AcpacC* strain showed a significant reduction in the rotten colonized area relative to that of the WT strain (Figures 5A,B and Supplementary Figures S3A,B). Four days after inoculation, Δ*AcpacC* strain showed an inhibition of the rotten area in nectarines and grape berries by up to 47 and 26%, respectively, compared with the WT strain (Figure 5B and Supplementary Figure S3B). The virulence was reverted by the complemented *AcpacC-c* strain (Figure 5A). The analysis of OTA accumulation in the infected nectarine tissue 5 days after inoculation revealed a three-fold reduction in OTA synthesis by the mutant strain, compared with the WT strain (Figure 5C); at the same time point, no OTA accumulation was detected by the Δ*AcpacC* in the inoculated grape berries (Supplementary Figure S3C). Nectarines infected with the Δ*AcpacC* mutant showed a 2–10 fold down-regulation of the transcript levels of all the five genes involved in OTA biosynthesis, compared with infections with the WT strain (Figure 5D), suggesting that AcPacC is essential for OTA production and probably directly involved in regulating transcription of the genes in OTA biosynthetic pathway. One would expect that reduction in OTA production by Δ*AcpacC* mutant may contribute to a reduction in virulence in this strain, however, in this regard, it should be noted that pathogenicity of the *A. carbonarius pks* mutant, which is unable to produce OTA, remained very similar to that of the WT (data not shown).

**FIGURE 6:**
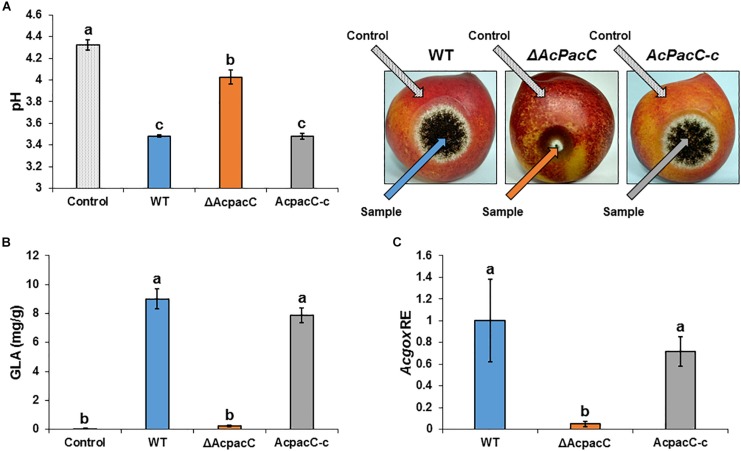
Effects of AcPacC on GLA production in colonized nectarines. pH of nectarine tissues **(A)**, GLA accumulation **(B)**, and *Acgox* relative expression **(C)** were measured in fruits infected with the WT, Δ*AcpacC* and *AcpacC-c* strains at day 4 post-inoculation. Error bars represent standard error of three independent biological replicates. Different letters above the columns indicate statistically significant differences at *p* < 0.05, as determined using the Tukey’s honest significant difference test.

To gain an understanding of the potential mechanism underlying the reduced pathogenicity of the Δ*AcpacC* strain, the mutant was assessed for several physiological characteristics that have previously been associated with virulence in this pathogen. It has been proposed that one of the factors that contribute to pathogenicity of *A. carbonarius* is its ability to reduce the pH of infected grape tissue through the production of gluconic acid (Maor et al., 2017). Indeed, 4 days after inoculation, colonization of nectarine tissue by *A. carbonarius* WT strain reduced pH from 4.3 in the healthy part of the fruit to 3.5 in the decayed tissue (Figure 6A). This further acidification of the rotten tissue was accompanied by an accumulation of 9 mg/g of gluconic acid (Figure 6B). In contrast, the Δ*AcpacC* strain showed a smaller reduction in pH and resulted in the accumulation of minimum amount of the gluconic acid (0.24 mg/g; Figures 6A,B). Gene expression analysis in the tissue inoculated with the Δ*AcpacC* mutant showed a 10-fold down-regulation of *Acgox* expression, which may explain poor gluconic acid formation *in vivo* (Figure 6C). Thus, our data indicate that AcPacC is required for *A. carbonarius* virulence in fruits, most likely by the regulation of the expression of the *gox* gene.

Disruption of *pacC* resulted in reduced pathogenicity of *P. expansum* in pear and apple fruits through mediating a virulence factor glucose oxidase (Chen et al., 2018). In that study, glucose oxidase was identified as alkaline-expressed protein by proteome analysis and proved to be involved in the virulence of *P. expansum*. These results confirmed the findings of an earlier study of Barad et al. (2014), where *P. expansum pacC*-RNAi mutants reduced gluconic acid (which is regulated by *gox* expression) and patulin accumulation in apples and showed a 45% reduction in fungal pathogenicity, compared to the WT.

Furthermore, since *Aspergillus* enzymes are involved in degradation of plant cell wall polysaccharides, the expression levels of four genes encoding polygalacturonase, pectate lyase, cellulase and hemicellulase were also analyzed during fruit colonization, and all of them were down-regulated in the Δ*AcpacC* strain compared with the WT (Supplementary Figure S4). This down-regulation suggests possible involvement of AcPacC in the regulation of the cell wall-degrading enzymes during fruit colonization. Zhang et al. (2013) have reported similar findings, where PdPacC has been shown to be important for the pathogenicity of *P. digitatum* in citrus fruits via regulation of polygalacturonase and the pectin lyase genes. In *Colletotrichum gloeosporioides*, *pac1* mutants showed reduction of *pelb* gene expression level, with consequent delayed pectate lyase secretion and dramatically reduced virulence in avocado fruits (Miyara et al., 2008). Overall, our results suggest that AcPacC may contribute to the pathogenesis of *A. carbonarius* through regulating different PacC-dependent genes or pathways involved in virulence.

## Conclusion

In this study we demonstrated that disruption of the pH signaling transcription factor PacC significantly decreased the virulence of *A. carbonarius* on deciduous fruits. This phenotype is associated with an impairment in fungal growth, decreased accumulation of gluconic acid and reduced synthesis of pectolytic enzymes. We showed that glucose oxidase-encoding gene, which is essential for gluconic acid production and acidification during fruit colonization, was significantly down-regulated in the Δ*AcpacC* mutant, suggesting that *gox* is PacC-responsive gene. Recently we have provided evidence that deletion of *gox* gene in *A. carbonarius* led to a reduction in virulence toward nectarine and grape fruits (data not shown), further indicating that GOX is a virulence factor of *A. carbonarius*, and its expression is regulated by PacC. The deletion of *AcpacC* may also affect the pathogenesis of *A. carbonarius* through the down-regulation of the cell wall-degrading enzymes such as polygalacturonase, pectate lyase, cellulase and hemicellulose. It is also clear from the present data that PacC in *A. carbonarius* is a key factor for the biosynthesis of secondary metabolites, such as OTA. Additional work is needed in order to gain a genomic perspective of the function of PacC during pathogenesis. Therefore, comparison of the transcriptomes of the WT and the Δ*AcpacC* mutant during fruit infection would contribute for the better understanding of the molecular regulatory network in pathogenicity and OTA biosynthesis of *A. carbonarius*.

## Data Availability Statement

All datasets generated for this study are included in the article/Supplementary Material.

## Author Contributions

OB, DP, YB, and ES conceived and designed the experiments. OB, UM, SS, and VZ performed the experiments. OB, SS, DP, YB, and ES analyzed the data. OB, DP, and ES wrote the manuscript. All authors read and approved the final manuscript.

## Conflict of Interest

The authors declare that the research was conducted in the absence of any commercial or financial relationships that could be construed as a potential conflict of interest.
