# Supplementary material for: The pH-Responsive Transcription Factor PacC Governs Pathogenicity and Ochratoxin A Biosynthesis in Aspergillus carbonarius
Source: Front Microbiol. 2020 Feb 13;11:210. doi: 10.3389/fmicb.2020.00210 (PMC7031272; doi:10.3389/fmicb.2020.00210)
Supplement: Supplementary file 1 [file Data_Sheet_1.docx]

Supplementary Material

Figures and tables

# Supplementary tables

**Table S1. List of primers used in the study** **to create and confirm the mutant and complement strains**

| **#** | **Primer name** | **Sequence** | **Description** |
| --- | --- | --- | --- |
| 1 | U-f1 | GGGTTTAAUCGCTGACTCTTGACGAACAG | Amplification of *AcpacC* promoter for construction of knockout vector |
| 2 | U-r1 | GGACTTAAUTCACCCACCAACCATCAAC |  |
| 3 | D-f1 | GGCATTAAUGCTTCTCCTCTTTCAGCTTGTC | Amplification of *AcpacC* terminator for construction of knockout vector |
| 4 | D-r1 | GGTCTTAAUAGACGAGACGATGACGCTTC |  |
| 5 | H-f1 | AGCTGCGCCGATGGTTTCTACAA | Amplification of hygromycin resistance gene internal sequence for verification |
| 6 | H-r1 | GCGCGTCTGCTGCTCCATACAA |  |
| 7 | P-f1 | TTTGAGCCCTCGTTGAACC | Amplification of *AcpacC* internal sequence for verification |
| 8 | P-r1 | TGGTGTCCTGCATCTGCTG |  |
| 9 | F-f1 | ATGGGTGATCAACTCCGTAAG | Flanking the *AcpacC* recombination sites for verification |
| 10 | F-r1 | TGGCTGACTGATGCAGACTC |  |
| 11 | RFC-f1 | TGACTTACCTATTCTACCCAAGCATCGATATGGCCAAGTTGACCAGTG | RF cloning of phleomycin resistance gene for construction of complementation vector |
| 12 | RFC-r1 | TGGATCCCGGTCGGCATCTACTTCAGTCCTGCTCCTCGGC |  |
| 13 | U-f1 | GGGTTTAAUCGCTGACTCTTGACGAACAG | Amplification of the *AcpacC* cassette for construction of complementation vector |
| 14 | U-r2 | GGACTTAAUGCGTGCAGTTTAGTGGCTC |  |

**Table S2. Primer sequences used for qRT-PCR analysis**

| **#** | **Primer name** | **Sequence** | **Description** |
| --- | --- | --- | --- |
| 1 | Btub_carF | GGACGAGATGGAGTTCACTGA | *β-tubulin* control gene |
| 2 | Btub_carR | CCTCTTGCTCAAGGACCTCCT |  |
| 3 | Acgox f2 | TCGAACACTCTGGCATTGGA | Glucose oxidase (*gox*) gene |
| 4 | Acgox r2 | GTGGTTTGGTCCTGCAGGTT |  |
| 5 | bZip_carF | CTCGACGGTTCGAGCCTTCT | OTA cluster bZip transcription factor |
| 6 | bZip_carR | GCATTCGCTCTAGCTGCTCGA |  |
| 7 | HAL_carF | GCCAGTAGAGGGACAGCCAT | OTA cluster halogenase |
| 8 | HAL_carR | GCTGGAGGTGGTGGTTGAGA |  |
| 9 | P450_carF | CCATCGTCTCCAGAGAATCAGT | OTA cluster cytochrome P450 monooxygenase |
| 10 | P450_carR2 | GGTCTCGTCGTGATGAATCAAG |  |
| 11 | NRPS_carF | CGGTAGAAAGACTGCAGTCCAT | OTA cluster non-ribosomal peptide synthetase |
| 12 | NRPS_carR | CGTCGGAATCCATTGCGCTGA |  |
| 13 | PKS_carF | GGGATCGTACGATCTGGTGAT | OTA cluster polyketide synthase |
| 14 | PKS_carR | GGGAACACATGAGGTCAGGCT |  |
| 15 | AccbhB f1 | GAGCGACGACAGCAATTATGAG | Cellobiohydrolase B (*cbhB*) gene |
| 16 | AccbhB r1 | AAGGGAGGTTGGAGACATCCA |  |
| 17 | AcxynB f1 | GTGGTCCGACGTGAGCAACT | Xylanase B (*xynB*) gene |
| 18 | AcxynB r1 | ATAGGTGATGTTCTGGGCACTTC |  |
| 19 | AcpelA f1 | GTGGTGACGCTATCACTCTTGATG | Pectin lyase A (*pelA*) gene |
| 20 | AcpelA r1 | CCGATGCGAGCGGTAGTAAC |  |
| 21 | AcpgaC f1 | GGACACCAAGGGCAGCAA | Polygalacturonase C (*pgaC*) gene |
| 22 | AcpgaC r1 | AGATCTTCAGGCCGGTGATG |  |

# Supplementary figures


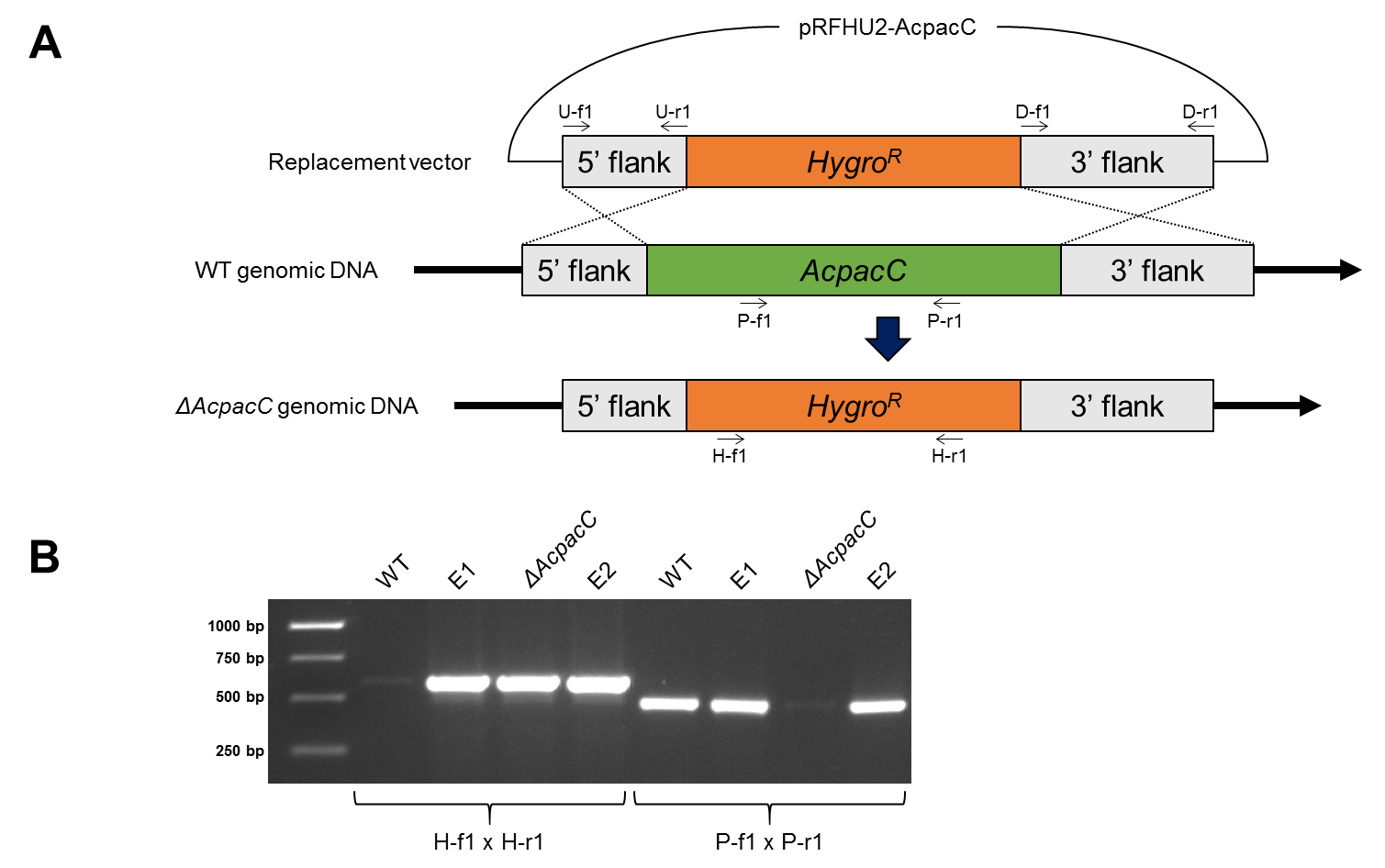


**FIGURE S1: Generation of *ΔAcpacC* knockout mutant.** **(A)** Schematic representation to scale of the deletion of *AcpacC*. The gene replacement vector pRFHU2-AcpacC was constructed by cloning the 5' and 3' flanking regions on each side of the hygromycin resistance gene *hph*. **(B)** Verification of the positive transformants by PCR analysis. Primer pairs P-f1 x P-r1 and H-f1 x H-r1 were used to verify the removal of the *AcpacC* ORF and the acquisition of the *hph* ORF in the deletant strain respectively. Primers for amplification of the flanking fragments and verification of the positive transformants are listed in supplementary table S1. E1 and E2, ectopic colonies that incorporated the replacement vector outside the desired genomic loci.


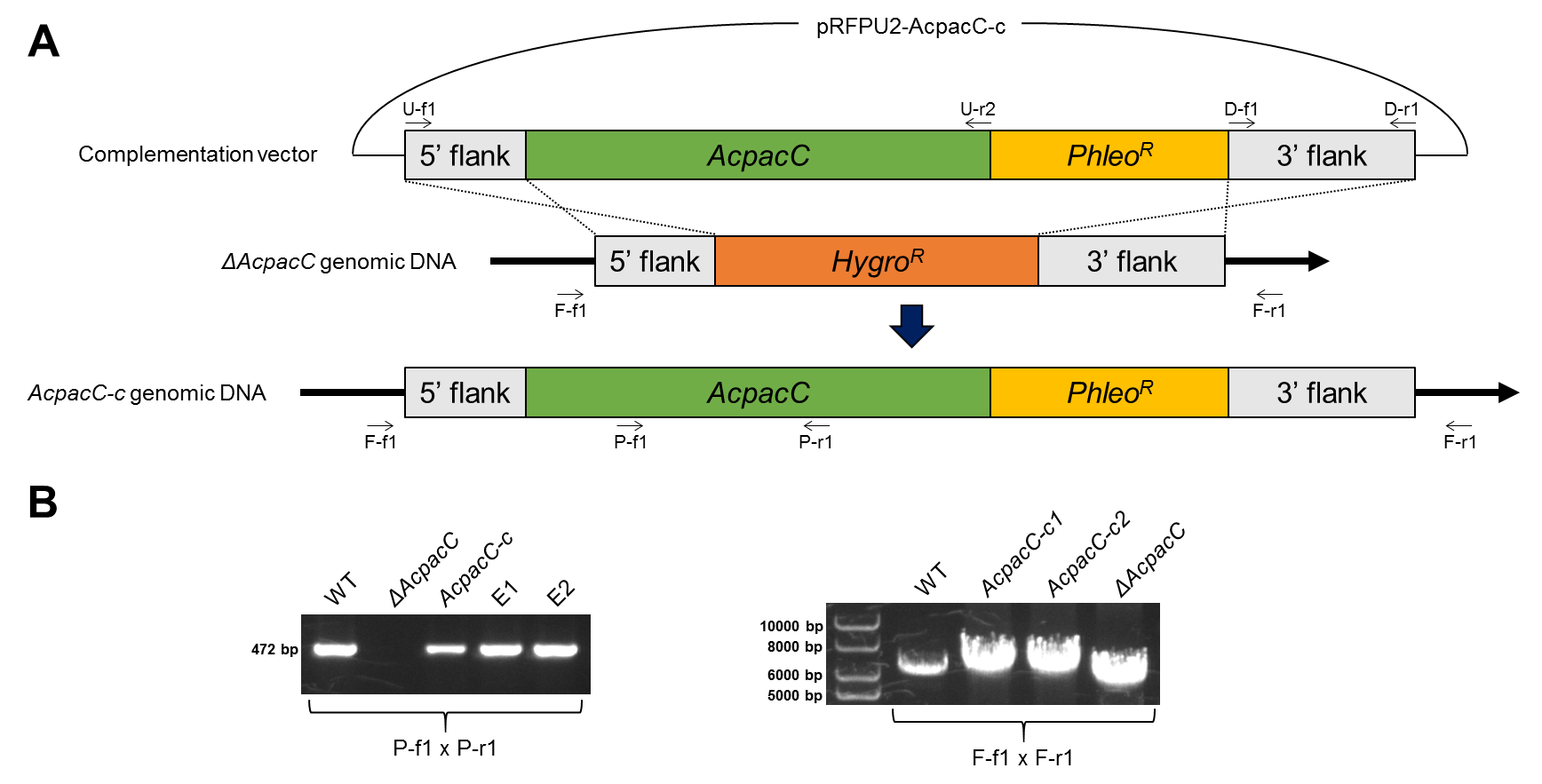


**FIGURE S2: Generation of *AcpacC-c* complementation strain.** **(A)** Schematic representation to scale of the genetic construct for *AcpacC* complementation. The complementation vector was constructed by cloning the entire *AcpacC* cassette and 3' flanking region on each side of the phleomycin resistance gene *ble* to yield the vector pRFPU2-AcpacC-c. **(B)** Verification of the positive transformants by PCR analysis. Primer pairs P-f1 x P-r1 and F-f1 x F-r1 were used to verify the reintroduction of the *AcpacC* ORF and to check for the correct genomic size following recombination respectively. Primers for the construction of the vector and verification of the positive transformants are listed in supplementary Table S1.


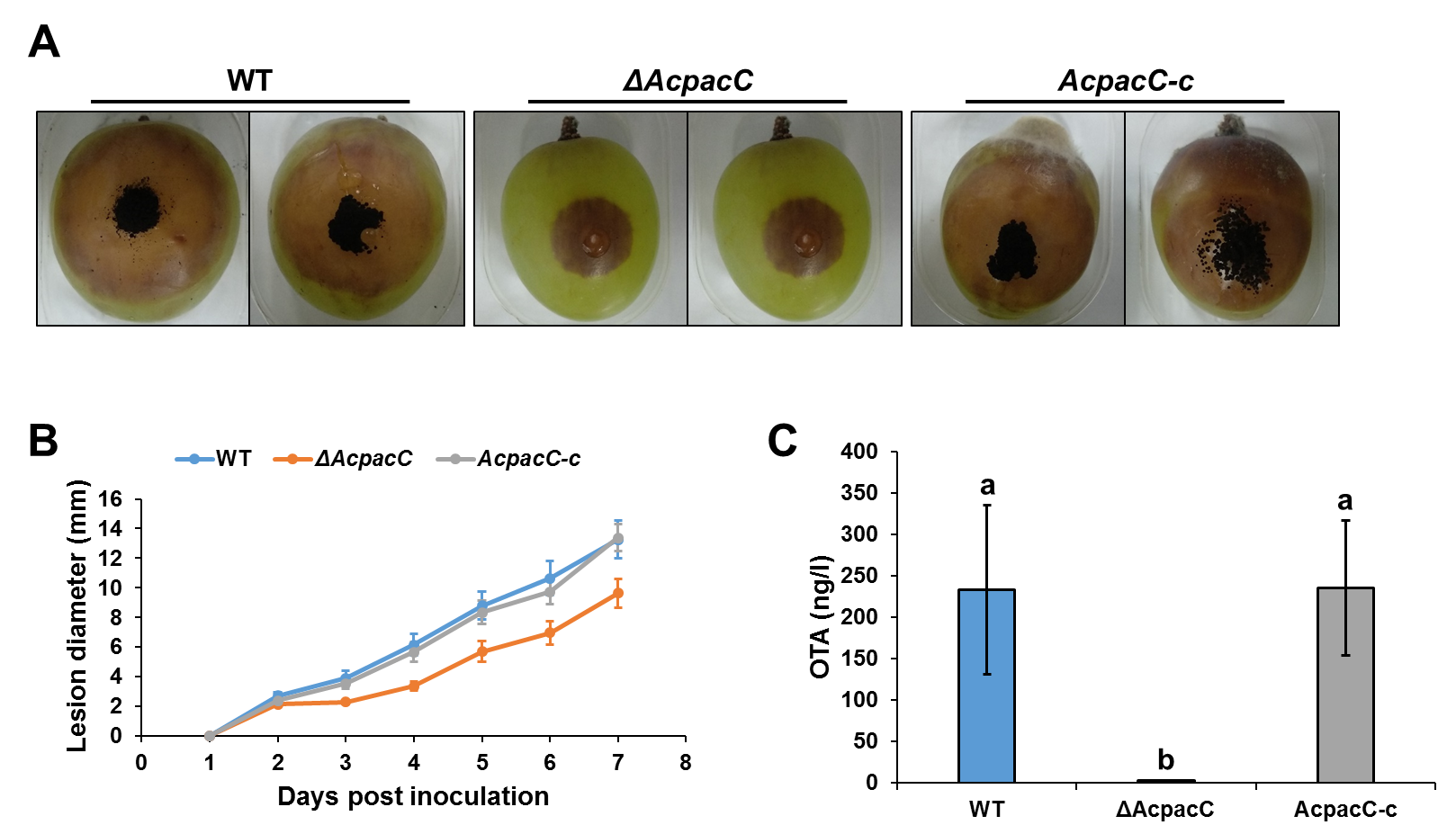


**FIGURE S3: PacC is important for *A. carbonarius* virulence and OTA production in grapes.** **(A)** Visualization of 'Zani' seedless grape berries inoculated with spore suspensions of the WT, *ΔAcpacC* and *AcpacC-c* strains of *A. carbonarius*. **(B)** Rot diameter on grape berries. **(C)** OTA accumulation in 'Zani' seedless grape berries. OTA accumulation was analyzed at 5 days post-inoculation. All measurements were repeated on 10 to 12 grape berries, error bars represent the standard error of the mean (SEM) across three technical replicates. Different letters above the columns indicate statistically significant differences at p<0.05, as determined using the Tukey's honest significant difference test.


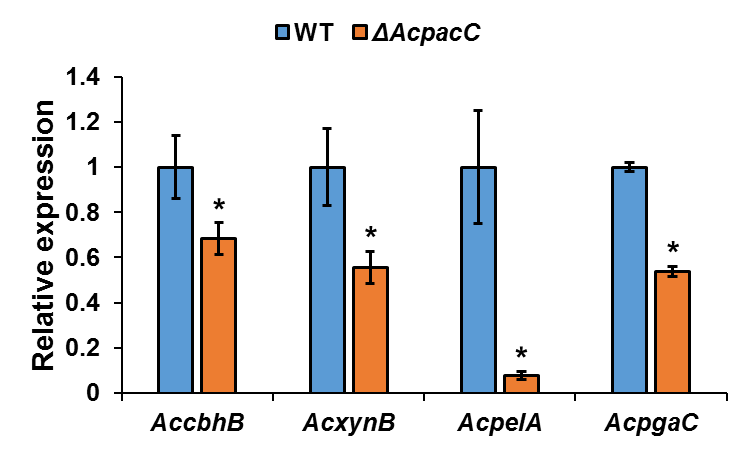


**FIGURE S4.** **PacC controls the expression of cell-wall degrading enzymes in *A. carbonarius*.** Relative expression of cell-wall degrading genes in WT and *ΔAcpacC* strains. RNA was extracted from infected nectarines 4 days post inoculation. Error bars represent standard error of three independent biological replicates. Asterisks denote significant differences between strains at p<0.05.
